# Sarcopenia as an Independent Risk Factor for Decreased BMD in COPD Patients: Korean National Health and Nutrition Examination Surveys IV and V (2008-2011)

**DOI:** 10.1371/journal.pone.0164303

**Published:** 2016-10-17

**Authors:** Dong-Won Lee, Eun-Young Choi

**Affiliations:** 1 Division of Pulmonology, Department of Internal Medicine, Andong Sungso Hospital, Andong, Korea; 2 Division of Pulmonology and Allergy, Department of Internal Medicine, Yeungnam University Hospital, Daegu, Korea; University of Nevada Las Vegas, UNITED STATES

## Abstract

**Background:**

A decrease in bone mineral density (BMD) is a systemic consequence of chronic obstructive pulmonary disease (COPD). Past reports have rarely examined any correlation between sarcopenia and BMD. We investigated the relationship cross-sectionally between the presence of sarcopenia and BMD reduction in COPD patients.

**Methods:**

COPD patients aged 50 or older with qualifying spirometry and dual-energy X-ray absorptiometry data were from participants in the Korean National Health and Nutrition Examination Surveys IV and V (2008–2011).

**Results:**

There were 286 (33.3%) subjects in the sarcopenia group and 572 (66.7%) in the non-sarcopenia group. The sarcopenia group had lower T-scores than the non-sarcopenia group (femur: -0.73±0.88 vs. -0.18±0.97, p < 0.001; femur neck: -1.44±0.98 vs. -0.99±1.06, p < 0.001; lumbar: -1.38±1.36 vs. -0.84±1.38, p < 0.001). The prevalences of osteopenia and osteoporosis were 60.8% and 22.0%, respectively, in the sarcopenia group and 45.6% and 13.3% in the non-sarcopenia group (both p < 0.001). After adjusting for multiple variables, the presence of sarcopenia associated with increased the risk of osteopenia, osteoporosis, and a low BMD (OR = 3.227, 95% CI = 2.125–4.899, p < 0.001, OR = 6.952, 95% CI = 3.418–14.139, p < 0.001, and OR = 3.495, 95% CI = 2.315–5.278, p < 0.001, respectively). In a subgroup analysis, similar OR changes were confirmed in the high-body-weight group (*n* = 493) (OR = 2.248, 95% CI = 1.084–4.665, p = 0.030, OR = 4.621, 95% CI = 1.167–18.291, p = 0.029, and OR = 2.376, 95% CI = 1.158–4.877, p = 0.018, respectively).

**Conclusions:**

The presence of sarcopenia was associated with increased the risk for decreased BMD in COPD.

## Introduction

Chronic obstructive pulmonary disease (COPD) is highly prevalent worldwide. Reported as the sixth leading cause of death in 1990, it is now ranked the fourth leading cause of death, and is projected to rank third by 2020 [1]. Limited progressive chronic airflow is a typical feature of COPD [2]. In addition, it is associated with extrapulmonary comorbidities such as cardiovascular disease, decreases in bone mineral density (BMD), decreases in skeletal muscle mass (SM), and deterioration in muscle strength, which can also negatively affect health outcomes [3]. The increase in various comorbidities damages functional status, degrades the quality of life, and increases mortality [4]. Of these comorbidities, osteoporosis is a major problem that requires therapeutic intervention [5].

In the general population, risk factors for osteoporosis include female sex, age, low body weight, chronic glucocorticoid use, and endocrinopathies such as hyperthyroidism and primary hyperparathyroidism [6–8]. More recently, a decrease in skeletal muscle, or sarcopenia, has been discovered as a risk factor [9]. The major underlying risk factor for osteoporosis in COPD is not yet clearly understood, but factors such as old age, female sex, low body weight, and body mass index (BMI) are associated with a decrease in BMD in COPD patients [10, 11]. Compared to SM, body weight and BMI have limitations in accurately reflecting body composition. In a study on aging and body composition, the prevalences of sarcopenia were 8.9% in the overweight (BMI = 25–29) group and 7.1% in the obese (BMI >30) group [12, 13]. Overweight to obese but otherwise healthy people frequently have sarcopenia, and the prevalence may increase further in COPD patients. Known body indexes associated with osteoporosis risk factors in COPD patients are low body weight and a low BMI [14–16]. Sarcopenia, a major complication of COPD, is frequently observed even without low body weight or low BMI. Nevertheless, few studies on the relationship between sarcopenia and BMD have been published.

In this study, the relationship between the presence of sarcopenia and decreased BMD in COPD patients was investigated using data from the Korean National Health and Nutrition Examination Survey (KNHANES) IV and V conducted from 2008 to 2011.

## Subjects and Methods

### Study population

This study was based on data acquired from KNHANES, which has been conducted periodically by the Division of Health and Nutritional Survey at the Korean Centers for Disease Control and Prevention (KCDCP) since 1998. It is an ongoing, population-based, cross-sectional, and nationally representative survey, and it provides the largest publicly available database in South Korea. Participants were chosen from candidates using a proportional-allocation, systematic sampling with multistage stratification (by age, gender, and region). The KNHANES consists of three different components: a health interview, a nutrition survey, and a health examination. Data were collected by household interviews and direct standardized physical examinations were conducted at mobile examination centers.

Written informed consent was provided by all participants. The protocols for KNHANES IV and V were approved by the institutional review board of the KCDC (2008-04EXP-01-C, 2009-01CON-03-2C, 2010-02CON-21-C, 2011-02CON-06-C). The current study did not require additional institutional review board approval because the KNHANES dataset is publicly available.

**Fig 1:**
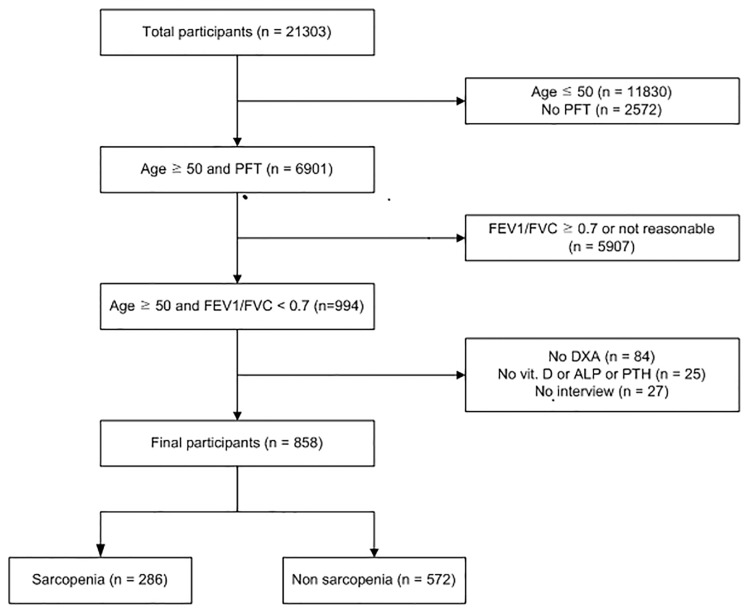
Flow chart.

From July 2008 to May 2011, KNHANES interviews, surveys, and examinations were completed in 21,303 individuals. Of them, our study included 858 subjects aged 50 or older, who underwent qualified spirometry and DXA, and were COPD patients, defined by a forced expiratory volume in 1 s (FEV_1_) / forced expiratory vital capacity (FVC) < 0.7 (Fig 1).

### Measurements

Trained interviewers administered a standardized questionnaire to the study subjects on their medical history, smoking frequency, expression of dyspnea, number of days with dyspnea, and physical inactivity level. Blood samples were taken from them after at least 8 h fasting. Blood samples were collected in serum separator tubes. Plasma was separated by centrifugation and stored at -70°C. Serum levels of ALP were measured using an automatic analyzer (Hitachi 7600 automatic analyzer; Hitachi, Tokyo, Japan). Total serum 25-hydroxyvitamin D (25-OHD) levels were measured using radioimmunoassay kits (Diasorin, Stillwater, MN, USA), and expressed in ng/mL (conversion factor, 2.5 for nmol/L). Plasma PTH was measured by the N-tact PTH assay using the LIAISON (Diasorin) chemiluminescence immunoassay method, and expressed in pg/mL.

Their health-related quality of life (QOL) was measured using a validated Korean version of the five-item self-administered EuroQOL instrument (EQ-5D). EQ-5D reports current health status and has a descriptive system and a visual analog scale (VAS). The descriptive assessments consist of five items with three possible answers for each. The assessments covered five dimensions relative to QOL: mobility, self-care, usual activities, pain/discomfort, and anxiety/depression. Each item can be used to represent the health status profiles of the subjects or can be converted into a summary index (the EQ-5D index). VAS is a measurement scale ranging from 0 (indicating the worst health status) to 100 (indicating the best health status). Height and body weight were measured during the participants’ visits to the examination center, clad in disposable examination gowns.

### Lung function measurements

Trained technicians performed spirometry according to the 1994 American Thoracic Society recommendations using the same type of dry rolling-seal spirometer (model 2130; Sensor Medics, Yorba Linda, CA, USA) for all of the subjects. Spirometry was repeated at least three times and up to a maximum of eight times to ensure the reproducibility and validity of results. The flow volume curves were unacceptable in the presence of a cough, glottis closure, early termination, leak, or variable effort. The spirometry results were transmitted to the central quality control center to ensure that they satisfied the acceptability and reproducibility criteria.

### Dual energy X-ray absorptiometry measurements

Body composition was analyzed with whole-body DXA, which can distinguish among three body components (fat mass, bone minerals, and fat-free soft tissue) using different X-ray attenuation properties. This survey used the bone density regulator Discovery fan beam densitometer (Hologic Inc., Bedford, MA, USA) and the Hologic Discovery software (ver. 13.1). The precision of the DXA examinations was assessed, and the central quality control center reviewed and analyzed all of the results.

### Definitions

A COPD patient was defined as one who had a FEV_1_/FVC < 0.7 by spirometry. The WHO criteria define osteopenia and osteoporosis as conditions with a spine or hip BMD T-score of -1 to -2.5, and a T-score of -2.5 or lower by DXA. A low BMD was defined as a spine or hip BMD T-score of -1 or lower. BMI was defined as weight (kg) divided by height in meters squared (m^2^). Appendicular skeletal muscle mass (ASM) was defined as the ASM less the appendicular bone mass, and the ASM mass index (ASMI) was defined as ASM (kg) divided by the height in meters squared (m^2^). According to the recommendation of the Asia Working Group for Sarcopenia (AWGS), sarcopenia was diagnosed when the ASMI by DXA was ≤ 7.0 kg/m^2^ for male patients and ≤ 5.4 kg/m^2^ for female patients [17]. According to the BMI Asia classification, a person with a BMI < 18.5 is classified as underweight, 18.5 to less than 23, normal, 23 to less than 25, overweight, and more than 25, obese. For the sub-analyses, a high body weight was defined as a BMI of 23 or higher, and a low body weight as a BMI less than 23.

### Statistical analysis

To compare the characteristics of the non-sarcopenia and sarcopenia groups, a T-test was conducted and the results are shown as means ± standard deviations. A χ^2^ test was also conducted to assess gender, BMI level, FEV_1_ (%) stage, and prevalence of osteopenia and osteoporosis. Correlation analyses were conducted to confirm the correlation between multiple variables and the T-score by area. To investigate the relationship between the T-score by body area and ASMI, factors such as age, gender, height, smoking frequency, FEV_1_ (%), level of blood vitamin D, parathyroid hormone (PTH) level, and alkaline phosphatase (ALP) level were corrected in multiple linear regression analyses. To confirm the effects of the weight, BMI, and ASMI on a low BMD, age, gender, height, smoking frequency, FEV_1_ (%), and levels of blood vitamin D, PTH, ALP, and physical inactivity were corrected in multivariate logistic analyses. To compare the risks of osteopenia, osteoporosis, and a low BMD according to the presence of sarcopenia, multivariate logistic analyses were conducted using models where the age, gender, height, weight, BMI, smoking frequency, FEV_1_ (%), levels of blood vitamin D, PTH, and ALP, and physical inactivity level factors were corrected. To compare the risks of osteopenia, osteoporosis, and a low BMD in the high body weight and low body weight groups according to the presence of sarcopenia, multivariate logistic analyses were conducted using models where the age, gender, height, smoking frequency, FEV_1_ (%), levels of blood vitamin D, PTH, and ALP, and physical inactivity level factors were corrected. The SPSS software (ver.18.0) was used for the analyses. Statistical significance was accepted when the P value was < 0.05.

## Results

### Group characteristics

In all, 858 patients were aged 50 or older and had a pulmonary function test result of FEV_1_/FVC < 0.7. There were 286 (33.3%) subjects in the sarcopenia group and 572 (66.7%) in the non-sarcopenia group. Those in the sarcopenia group were older and had a lower body weight and a lower BMI (*p* < 0.001); 23.5% of the subjects had a BMI of 23 or higher whereas 74.5% in the non-sarcopenia group had a BMI of 23 or higher (*p* < 0.001). Among all 858 patients, 493 had a BMI higher than 23; 13.5% of them had sarcopenia. The EQ-5D index of the sarcopenia group was lower than that of the non-sarcopenia group (*p* = 0.049), but in terms of the EQ-VAS, there were no significant differences. The sarcopenia group had more male than female subjects (P = 0.040). The level of physical inactivity of the sarcopenia group was higher than that of the non-sarcopenia group (*p* < 0.001). In all of the pulmonary function items, such as the FEV_1_ (L), FEV_1_ (%), and FEV_1_/FVC, the sarcopenia group had lower levels than the non-sarcopenia group (*p* < 0.001). In terms of blood vitamin D and PTH levels, which are associated with bone formation, no significant differences were observed between the groups (Table 1).

**Table 1:** Baseline characteristics of the sarcopenia and non-sarcopenia patients.

	Sarcopenia (*n* = 286) mean ± SD	Non-sarcopenia (*n* = 572) mean ± SD	P-value
Age (years)	67.37 ± 8.13	65.17 ± 7.63	< 0.001
Gender (male)	226 (79.0%)	415 (72.6%)	0.040
Height (cm)	162.58 ± 7.83	163.13 ± 8.84	0.350
Weight (kg)	56.72 ± 8.18	65.47 ± 9.74	< 0.001
BMI (kg/m^2^)	21.40 ± 2.27	24.52 ± 2.51	< 0.001
BMI			
Underweight (< 18.5)	33 (11.5%)	3 (0.5%)	< 0.001
Normal ≥ 18.5 and < 23	186 (65.0%)	143 (25.0%)	
Overweight ≥ 23 and < 25	52 (18.2%)	194 (33.9%)	
Obese > 25	15 (5.3%)	232 (40.6%)	
Smoking (pack years)	645.48 ± 446.40	604.23 ± 419.87	0.250
EQ-VAS score	75.51 ± 59.94	73.51 ± 19.02	0.998
EQ-5D index	0.89 ± 0.16	0.91 ± 0.15	0.049
Physical inactivity			< 0.001
Yes	88 (30.8%)	111 (19.4%)	
No	197 (68.9%)	460 (80.6%)	
Unknown	1 (0.3%)	0%	
Spirometry			
FEV_1_ (L)	2.09 ± 0.64	2.28 ± 0.65	< 0.001
FEV_1_ (%)	74.15 ± 17.45	78.68 ± 15.17	< 0.001
FVC (L)	3.39 ± 0.85	3.58 ± 0.92	0.003
FVC (%)	87.51 ± 15.18	90.27 ± 14.48	0.010
FEV_1_/FVC (%)	61.40 ± 8.23	63.64 ± 6.38	< 0.001
Vitamin D (ng/mL)	21.21 ± 8.33	20.93 ± 7.10	0.629
ALP (IU/L)	258.49 ± 77.99	244.92 ± 75.04	0.014
PTH (pg/mL)	68.18 ± 29.37	66.19 ± 25.19	0.304

SD, standard deviation; BMI, body mass index; EQ-5D, EuroQOL five-dimensions; EQ-VAS, EuroQOL visual analog scale; ALP, alkaline phosphatase; PTH, parathyroid hormone.

### Differences in BMD and the prevalence of osteopenia and osteoporosis in the sarcopenia group

**Fig 2:**
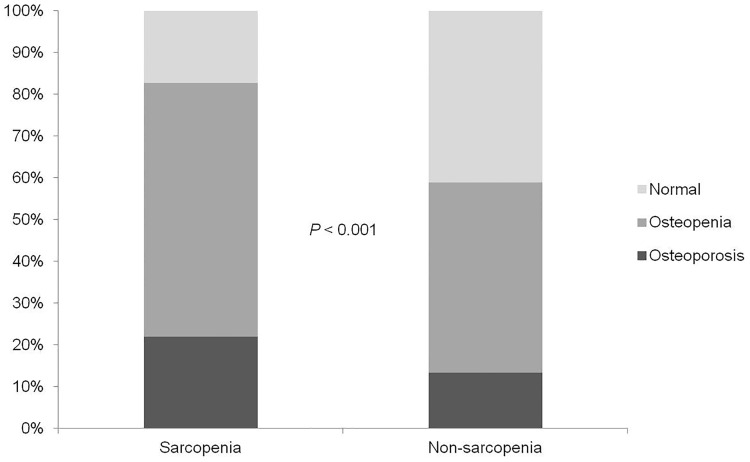
Prevalence of bone disease according to the sarcopenia group.

The sarcopenia group had lower T-scores than the non-sarcopenia group (*p* < 0.001; Table 2). The prevalences of osteopenia and osteoporosis were 60.8% and 22.0%, respectively, in the sarcopenia group and 45.6% and 13.3%, respectively, in the non-sarcopenia group (*p* < 0.001; Fig 2).

**Table 2:** T-score and prevalence of bone disease in each group.

	Sarcopenia (*n* = 286) mean ± SD	Non-sarcopenia (*n* = 572) mean ± SD	P-value
Femur T-score	-0.73±0.88	-0.18±0.97	< 0.001
Femur neck T-score	-1.44±0.98	-0.99±1.06	< 0.001
Lumbar T-score	-1.38±1.36	-0.84±1.38	< 0.001

SD, standard deviation

### Correlation between body mass index and T-score

Significant correlations were observed between T-scores and age, height, weight, BMI, and ASMI. ASMI had the second highest correlation with the scores, after body weight (*p* < 0.001; Table 3). In the models where age, gender, height, smoking frequency, levels of blood vitamin D, PTH, and ALP, and FEV_1_ (%) were adjusted for, weight, BMI, and ASMI showed relationships with the T-score. (*p* < 0.001; Table 4).

**Table 3:** Correlation analysis of body indexes for each T-score.

	Femur T-score		Femur neck T-score		Lumbar T-score	
	Coefficient	P-value	Coefficient	P-value	Coefficient	P-value
Age (years)	-0.351	< 0.001	-0.369	< 0.001	-0.198	< 0.001
Height (cm)	0.418	< 0.001	0.507	< 0.001	0.452	< 0.001
Weight (kg)	0.516	< 0.001	0.522	< 0.001	0.526	< 0.001
BMI (kg/m^2^)	0.332	< 0.001	0.265	< 0.001	0.320	< 0.001
ASMI (kg/m^2^)	0.503	< 0.001	0.519	< 0.001	0.462	< 0.001

BMI, body mass index; ASMI, appendicular skeletal muscle mass index.

**Table 4:** Multivariate linear regression analysis of body indexes for each T-score.

	B	SE	P-value	R2
Lumbar				
Weight (kg)	0.411	0.006	< 0.001	0.260
BMI (kg/m^2^)	0.328	0.017	< 0.001	0.262
ASMI (kg/m^2^)	0.268	0.065	< 0.001	0.208
Femur				
Weight (kg)	0.416	0.004	< 0.001	0.321
BMI (kg/m^2^)	0.329	0.011	< 0.001	0.321
ASMI (kg/m^2^)	0.373	0.042	< 0.001	0.313
Femur neck				
Weight (kg)	0.319	0.004	<0.001	0.305
BMI (kg/m^2^)	0.251	0.012	<0.001	0.305
ASMI (kg/m^2^)	0.284	0.045	<0.001	0.300

Adjusted for age, gender, height, smoking frequency, vitamin D, PTH and ALP levels, and FEV_1_ (%). B, standardized regression coefficient; SE, standard error; R2, adjusted R^2^; BMI, body mass index; ASMI, appendicular skeletal muscle mass index.

### Multivariate analyses of body indexes contributing to a low BMD

The correlations between various body indexes and a low BMD were investigated. Univariate analyses showed that weight, BMI, and ASMI were correlated with a low BMD (all *p* < 0.001). A multivariate analysis showed that weight, BMI, and ASMI were correlated with a low BMD after adjusting for factors such as age, gender, height, smoking frequency, level of blood vitamin D, PTH and ALP levels, FEV_1_ (%), and physical inactivity level (OR = 0.902, *p* < 0.001, OR = 0.755, *p* < 0.001, and OR = 0.438, *p <* 0.001, respectively). In subgroup analyses, similar OR changes were confirmed in both body weight groups (Table 5).

**Table 5:** Multivariate regression analysis of body indexes contributing to a low BMD.

	Total			High body weight group			Low body weight group		
	OR	*P*	R^2^	OR	*P*	R^2^	OR	*P*	R^2^
Weight	0.902	< 0.001	0.333	0.941	0.022	0.272	0.821	<0.001	0.267
BMI (kg/m^2^)	0.755	< 0.001	0.331	0.848	0.024	0.271	0.581	<0.001	0.266
ASMI (kg/m^2^)	0.438	< 0.001	0.303	0.659	0.030	0.270	0.455	0.004	0.220

Adjusted for age, gender, height, smoking frequency, vitamin D, PTH and ALP levels, FEV_1_ (%) and physical inactivity level.

OR, odds ratio: CI, confidence interval: R^2^, adjusted R^2^; BMI, body mass index; ASMI, appendicular skeletal muscle mass index.

### Odds ratios of osteopenia, osteoporosis, and a low BMD in the sarcopenia group

The effects of sarcopenia on the risks of osteopenia and osteoporosis were investigated. In the model corrected for age, gender, height, smoking frequency, levels of blood vitamin D, PTH, ALP, FEV_1_ (%), and physical inactivity level, the presence of sarcopenia increased the risk of osteopenia, osteoporosis, and a low BMD (OR = 3.227, 95% CI = 2.125–4.899, *p* < 0.001, OR = 6.952, 95% CI = 3.418–14.139, *p* < 0.001, and OR = 3.495, 95% CI = 2.315–5.278, *p* < 0.001, respectively). After adjusting for weight and BMI, the presence of sarcopenia still increased the risk of osteopenia and low BMD (OR = 1.822, 95% CI = 1.123–2.953, *p* = 0.015 and OR = 1.830, 95% CI = 1.133–2.956, *p* = 0.014, respectively; Table 6).

**Table 6:** Odds ratios of osteopenia, osteoporosis, and a low BMD by sarcopenia.

	Osteopenia			Osteoporosis			Low BMD		
	OR	95% CI	*P*	OR	95% CI	*P*	OR	95% CI	*P*
Model 1									
Non-sarcopenia	1			1			1		
Sarcopenia	3.227	2.125–4.899	< 0.001	6.952	3.418–14.139	< 0.001	3.495	2.315–5.278	< 0.001
Model 2									
Non-sarcopenia	1			1			1		
Sarcopenia	1.805	1.113–2.929	0.017	1.675	0.736–3.812	0.219	1.820	1.126–2.941	0.014
Model 3									
Non-sarcopenia	1			1			1		
Sarcopenia	1.827	1.126–2.964	0.015	1.736	0.764–3.942	0.187	1.837	1.136–2.969	0.013
Model 4									
Non-sarcopenia	1			1			1		
Sarcopenia	1.822	1.123–2.953	0.015	1.657	0.728–3.770	0.229	1.830	1.133–2.956	0.014

Model 1: Age, gender, height, smoking frequency, vitamin D, ALP and PTH levels, FEV_1_ (%), physical inactivity level. Model 2: Model 1 plus weight. Model 3: Model 1 plus BMI. Model 4: Model 1 plus weight and BMI. BMD, bone mineral density; OR, odds ratio; CI, confidence interval; ALP, alkaline phosphatase; PTH, parathyroid hormone; BMI, body mass index.

Subgroup analyses were conducted to confirm the effects of sarcopenia on BMD in each body weight group. In the high-weight group (BMI ≥ 23), the presence of sarcopenia increased the risk of osteopenia, osteoporosis, and a low BMD (OR = 2.248, 95% CI = 1.084–4.665, *p* = 0.030, OR = 4.621, 95% CI = 1.167–18.291, *p* = 0.029, and OR = 2.376, 95% CI = 1.158–4.877, *p* = 0.018, respectively). Similar OR changes were identified in the low-weight group (OR = 2.301, 95% CI = 1.239–4.275, *p* = 0.008, OR = 3.580, 95% CI = 1.410–9.089, *p* = 0.007, and OR = 2.439, 95% CI = 1.324–4.496, *p* = 0.004. respectively; Table 7).

**Table 7:** Odds ratios of osteopenia, osteoporosis, and a low BMD by sarcopenia in each body weight group (subgroup analysis).

	Osteopenia			Osteoporosis			Low BMD		
	OR	95% CI	*P*	OR	95% CI	*P*	OR	95% CI	*P*
High-weight group									
Non-sarcopenia	1			1			1		
Sarcopenia	2.248	1.084–4.665	0.030	4.621	1.167–18.291	0.029	2.376	1.158–4.877	0.018
Low-weight group									
Non-sarcopenia	1			1			1		
Sarcopenia	2.301	1.239–4.275	0.008	3.580	1.410–9.089	0.007	2.439	1.324–4.496	0.004

Adjusted for age, gender, height, smoking frequency, vitamin D, ALP and PTH levels, FEV_1_ (%), and physical inactivity level. BMD, bone mineral density; AWGS, Asia Working Group for Sarcopenia; OR, odds ratio; CI, confidence interval; ALP, alkaline phosphatase; PTH, parathyroid hormone.

## Discussion

Sarcopenia was identified in 33.3% of our COPD patients. In the overweight to obese group, sarcopenia was observed in 13.5%, which is approximately double that of the general population. In COPD patients, the sarcopenia group had a lower T-score level and higher prevalences of osteopenia and osteoporosis than the non-sarcopenia group. In a multivariate regression analysis, ASMI showed a strong association with the T-scores of all body areas. In addition, the negative effects of the ASMI on a low BMD were more significant than those of weight and BMI. In the models where factors such as the age, gender, height, smoking frequency, levels of blood vitamin D, PTH, ALP, FEV_1_ (%), and physical inactivity level were adjusted, the adjusted probability of osteopenia, osteoporosis, and a low BMD were 2–7 times higher in the sarcopenia group than the non-sarcopneia. Even after adjusting for weight and BMI, the probability of osteopenia and a low BMD were 1.8 times higher in the sarcopenia group than the non-sarcopneia. In the sub-analysis according to BMI, the presence of sarcopenia had 2–4 times higher probability for osteopenia, osteoporosis, and a low BMD in both the low-weight and high-weight groups.

SM loss is one of the most important systemic consequences of COPD. In COPD, the limb muscles most clearly show changes in SM. Thus, many studies on the limb muscles have been conducted [18]. The ASMI is a representative index of limb muscle mass, and the AWGS has suggested that ASMI is the single most important indicator of Asian sarcopenia [17]. The ASMI has been discussed with interest in recent COPD studies as a sarcopenia index [19–21]. In a recent study, sarcopenia was highly prevalent even in overweight to obese COPD groups [20]. In our study, the prevalence of sarcopenia in the overweight to obese COPD group was double that of the healthy group, as reported in previous studies.

In COPD, low body weight and a low BMI have already been reported to increase the risk of a low BMD [14–16]. We sought to identify the effects of sarcopenia on BMD in COPD, and found that the presence of sarcopenia itself was an independent risk factor of a decreased BMD in both the low-weight and high-weight groups. Overweight to obese COPD patients with sarcopenia could be overlooked as low-risk patients for a low BMD. However, our study showed that they also may be considered a high-risk group. Our study indicated that the low-weight group with sarcopenia must be considered a very high-risk group. In previous studies, sarcopenia in the healthy group was confirmed to be associated with a low BMD, but those studies did not consider weight and BMI [22–24]. Furthermore, in one study on COPD, the ASM was not suggested to be a risk factor for low BMD [25], whereas in our study, the ASMI had a stronger association with low BMD than other body indexes. Moreover, the presence of sarcopenia was a risk factor for decreased BMD in both the low-weight and high-weight groups. This outcome corresponds to that of a previous study on COPD mortality: higher mortality in subjects with a low fat-free mass index (FFMI) than in those with a low BMI [26].

Decreases in SM and BMD are major systemic consequences of COPD. A complex mechanism is involved in these changes, and systemic inflammation is thought to be an important factor. Various cytokines are involved in the process, particularly, TNF-α and IL-6 [27, 28]. The presence of such systemic inflammation might have caused the outcomes of this study. However, cytokines could not be assessed in this retrospective study. This issue will be covered in future studies.

This study had several limitations. First, muscle strength was not considered a sarcopenia evaluation item. Muscle strength is an important factor differentiating pre-sarcopenia from sarcopenia [29]. Second, DXA data, which are accurate for confirming body composition, are challenging to apply to all patients in practice. The AWGS recently suggested the results of bioelectrical impedance analysis (BIA) as a criterion for body composition measurement in sarcopenia [17]. BIA can be conveniently measured in the clinical setting. In this cohort study, BIA data were not available, so the sarcopenia results of BIA measurements could not be compared. Third, this study was conducted using a cohort database of Asian (Korean) people. Considering the differences in the body compositions of Westerners and Asians, the effects on Westerners could not be confirmed in this study. This issue is expected to be addressed in future studies.

## Conclusions

Two-thirds of the COPD patients had sarcopenia. In the overweight to obese group, sarcopenia was observed in 13.5% of subjects. The sarcopenia group had a lower T-score and higher prevalences of osteopenia and osteoporosis than the non-sarcopenia group. In addition, the presence of sarcopenia was significantly associated with increased the risk for osteopenia, osteoporosis, and a low BMD in both the low-weight and high-weight groups. Thus, in the evaluation of COPD patients, the presence of sarcopenia should be considered an independent risk factor for low BMD.

## Supporting Information

S1 Table This is the baseline characteristics of the sarcopenia and non-sarcopenia patients. (DOCX)

S2 Table This is the T-score and prevalence of bone disease in each group. (DOCX)

S3 Table This is the correlation analysis of body indexes for each T-score. (DOCX)

S4 Table This is the multivariate linear regression analysis of body indexes for each T-score. (DOCX)

S5 Table This is the multivariate regression analysis of body indexes contributing to a low BMD. (DOCX)

S6 Table This is the odds ratios of osteopenia, osteoporosis, and a low BMD by sarcopenia. (DOCX)

S7 Table This is the odds ratios of osteopenia, osteoporosis, and a low BMD by sarcopenia in each body weight group (subgroup analysis). (DOCX)
