# Supplementary material for: Sarcopenia as an Independent Risk Factor for Decreased BMD in COPD Patients: Korean National Health and Nutrition Examination Surveys IV and V (2008-2011)
Source: PLoS One. 2016 Oct 17;11(10):e0164303. doi: 10.1371/journal.pone.0164303 (PMC5066961; doi:10.1371/journal.pone.0164303)
Supplement: S1 Table — (DOCX) [file pone.0164303.s001.docx]

**Table 1**. Baseline characteristics of the sarcopenia and non-sarcopenia patients

|  | Sarcopenia (*n* = 286)  mean ± SD | Non-sarcopenia (*n* = 572)  mean ± SD | P-value |
| --- | --- | --- | --- |
| Age (years) | 67.37 ± 8.13 | 65.17 ± 7.63 | < 0.001 |
| Gender (male) | 226 (79.0%) | 415 (72.6%) | 0.040 |
| Height (cm) | 162.58 ± 7.83 | 163.13 ± 8.84 | 0.350 |
| Weight (kg) | 56.72 ± 8.18 | 65.47 ± 9.74 | < 0.001 |
| BMI (kg/m²) | 21.40 ± 2.27 | 24.52 ± 2.51 | < 0.001 |
| BMI |  |  |  |
| Underweight (< 18.5) | 33 (11.5%) | 3 (0.5%) | < 0.001 |
| Normal ≥ 18.5 and < 23 | 186 (65.0%) | 143 (25.0%) |  |
| Overweight ≥ 23 and < 25 | 52 (18.2%) | 194 (33.9%) |  |
| Obese > 25 | 15 (5.3%) | 232 (40.6%) |  |
| Smoking (pack years) | 645.48 ± 446.40 | 604.23 ± 419.87 | 0.250 |
| EQ-VAS score | 75.51 ± 59.94 | 73.51 ± 19.02 | 0.998 |
| EQ-5D index | 0.89 ± 0.16 | 0.91 ± 0.15 | 0.049 |
| Physical inactivity |  |  | < 0.001 |
| Yes | 88 (30.8%) | 111 (19.4%) |  |
| No | 197 (68.9%) | 460 (80.6%) |  |
| Unknown | 1 (0.3%) | 0% |  |
| Spirometry |  |  |  |
| FEV_1_ (L) | 2.09 ± 0.64 | 2.28 ± 0.65 | < 0.001 |
| FEV_1_ (%) | 74.15 ± 17.45 | 78.68 ± 15.17 | < 0.001 |
| FVC (L) | 3.39 ± 0.85 | 3.58 ± 0.92 | 0.003 |
| FVC (%) | 87.51 ± 15.18 | 90.27 ± 14.48 | 0.010 |
| FEV_1_/FVC (%) | 61.40 ± 8.23 | 63.64 ± 6.38 | < 0.001 |
| Vitamin D (ng/mL) | 21.21 ± 8.33 | 20.93 ± 7.10 | 0.629 |
| ALP (IU/L) | 258.49 ± 77.99 | 244.92 ± 75.04 | 0.014 |
| PTH (pg/mL) | 68.18 ± 29.37 | 66.19 ± 25.19 | 0.304 |

SD, standard deviation; BMI, body mass index; EQ-5D, EuroQOL five-dimensions; EQ‑VAS, EuroQOL visual analog scale; ALP, alkaline phosphatase; PTH, parathyroid hormone.
