# Supplementary material for: Sarcopenia as an Independent Risk Factor for Decreased BMD in COPD Patients: Korean National Health and Nutrition Examination Surveys IV and V (2008-2011)
Source: PLoS One. 2016 Oct 17;11(10):e0164303. doi: 10.1371/journal.pone.0164303 (PMC5066961; doi:10.1371/journal.pone.0164303)
Supplement: S2 Table — (DOCX) [file pone.0164303.s002.docx]

**Table 2**. T-score and prevalence of bone disease in each group

|  | Sarcopenia (*n* = 286)  mean ± SD | Non-sarcopenia (*n* = 572)  mean ± SD | P-value |
| --- | --- | --- | --- |
| Femur T-score | -0.73±0.88 | -0.18±0.97 | < 0.001 |
| Femur neck T-score | -1.44±0.98 | -0.99±1.06 | < 0.001 |
| Lumbar T-score | -1.38±1.36 | -0.84±1.38 | < 0.001 |

SD, standard deviation
