# Supplementary material for: Sarcopenia as an Independent Risk Factor for Decreased BMD in COPD Patients: Korean National Health and Nutrition Examination Surveys IV and V (2008-2011)
Source: PLoS One. 2016 Oct 17;11(10):e0164303. doi: 10.1371/journal.pone.0164303 (PMC5066961; doi:10.1371/journal.pone.0164303)
Supplement: S3 Table — (DOCX) [file pone.0164303.s003.docx]

**Table 3**. Correlation analysis of body indexes for each T-score

|  | Femur T-score | |  | Femur neck T-score | |  | Lumbar T-score | |
| --- | --- | --- | --- | --- | --- | --- | --- | --- |
|  | Coefficient | P-value |  | Coefficient | P-value |  | Coefficient | P-value |
| Age (years) | -0.351 | < 0.001 |  | -0.369 | < 0.001 |  | -0.198 | < 0.001 |
| Height (cm) | 0.418 | < 0.001 |  | 0.507 | < 0.001 |  | 0.452 | < 0.001 |
| Weight (kg) | 0.516 | < 0.001 |  | 0.522 | < 0.001 |  | 0.526 | < 0.001 |
| BMI (kg/m²) | 0.332 | < 0.001 |  | 0.265 | < 0.001 |  | 0.320 | < 0.001 |
| ASMI (kg/m²) | 0.503 | < 0.001 |  | 0.519 | < 0.001 |  | 0.462 | < 0.001 |

BMI, body mass index; ASMI, appendicular skeletal muscle mass index.
