# Supplementary material for: Sarcopenia as an Independent Risk Factor for Decreased BMD in COPD Patients: Korean National Health and Nutrition Examination Surveys IV and V (2008-2011)
Source: PLoS One. 2016 Oct 17;11(10):e0164303. doi: 10.1371/journal.pone.0164303 (PMC5066961; doi:10.1371/journal.pone.0164303)
Supplement: S4 Table — (DOCX) [file pone.0164303.s004.docx]

**Table 4**. Multivariate linear regression analysis of body indexes for each T-score

|  | B | SE | P-value | R2 |
| --- | --- | --- | --- | --- |
| Lumbar |  |  |  |  |
| Weight (kg) | 0.411 | 0.006 | < 0.001 | 0.260 |
| BMI (kg/m²) | 0.328 | 0.017 | < 0.001 | 0.262 |
| ASMI (kg/m²) | 0.268 | 0.065 | < 0.001 | 0.208 |
| Femur |  |  |  |  |
| Weight (kg) | 0.416 | 0.004 | < 0.001 | 0.321 |
| BMI (kg/m²) | 0.329 | 0.011 | < 0.001 | 0.321 |
| ASMI (kg/m²) | 0.373 | 0.042 | < 0.001 | 0.313 |
| Femur neck |  |  |  |  |
| Weight (kg) | 0.319 | 0.004 | <0.001 | 0.305 |
| BMI (kg/m²) | 0.251 | 0.012 | <0.001 | 0.305 |
| ASMI (kg/m²) | 0.284 | 0.045 | <0.001 | 0.300 |

Adjusted for age, gender, height, smoking frequency, vitamin D, PTH and ALP levels, and FEV_1_ (%). B, standardized regression coefficient; SE, standard error; R2, adjusted R^2^; BMI, body mass index; ASMI, appendicular skeletal muscle mass index.
