# Supplementary material for: Sarcopenia as an Independent Risk Factor for Decreased BMD in COPD Patients: Korean National Health and Nutrition Examination Surveys IV and V (2008-2011)
Source: PLoS One. 2016 Oct 17;11(10):e0164303. doi: 10.1371/journal.pone.0164303 (PMC5066961; doi:10.1371/journal.pone.0164303)
Supplement: S5 Table — (DOCX) [file pone.0164303.s005.docx]

**Table 5**. Multivariate regression analysis of body indexes contributing to a low BMD

|  | Total | | |  | High body weight group | | |  | Low body weight group | | |  |
| --- | --- | --- | --- | --- | --- | --- | --- | --- | --- | --- | --- | --- |
|  | OR | *P* | R^2^ |  | OR | *P* | R^2^ |  | OR | *P* | R^2^ |  |
| Weight | 0.902 | < 0.001 | 0.333 |  | 0.941 | 0.022 | 0.272 |  | 0.821 | <0.001 | 0.267 |  |
| BMI (kg/m²) | 0.755 | < 0.001 | 0.331 |  | 0.848 | 0.024 | 0.271 |  | 0.581 | <0.001 | 0.266 |  |
| ASMI (kg/m²) | 0.438 | < 0.001 | 0.303 |  | 0.659 | 0.030 | 0.270 |  | 0.455 | 0.004 | 0.220 |  |

Adjusted for age, gender, height, smoking frequency, vitamin D, PTH and ALP levels, FEV_1_ (%) and physical inactivity level

OR, odds ratio: CI, confidence interval: R^2^, adjusted R^2^; BMI, body mass index; ASMI, appendicular skeletal muscle mass index.
