# Supplementary material for: Sarcopenia as an Independent Risk Factor for Decreased BMD in COPD Patients: Korean National Health and Nutrition Examination Surveys IV and V (2008-2011)
Source: PLoS One. 2016 Oct 17;11(10):e0164303. doi: 10.1371/journal.pone.0164303 (PMC5066961; doi:10.1371/journal.pone.0164303)
Supplement: S6 Table — (DOCX) [file pone.0164303.s006.docx]

**Table 6**. Odds ratios of osteopenia, osteoporosis, and a low BMD by sarcopenia

|  | Osteopenia | | |  | Osteoporosis | | |  | Low BMD | | |
| --- | --- | --- | --- | --- | --- | --- | --- | --- | --- | --- | --- |
|  | OR | 95% CI | *P* |  | OR | 95% CI | *P* |  | OR | 95% CI | *P* |
| Model 1 |  |  |  |  |  |  |  |  |  |  |  |
| Non-sarcopenia | 1 |  |  |  | 1 |  |  |  | 1 |  |  |
| Sarcopenia | 3.227 | 2.125–4.899 | < 0.001 |  | 6.952 | 3.418–14.139 | < 0.001 |  | 3.495 | 2.315–5.278 | < 0.001 |
| Model 2 |  |  |  |  |  |  |  |  |  |  |  |
| Non-sarcopenia | 1 |  |  |  | 1 |  |  |  | 1 |  |  |
| Sarcopenia | 1.805 | 1.113–2.929 | 0.017 |  | 1.675 | 0.736–3.812 | 0.219 |  | 1.820 | 1.126–2.941 | 0.014 |
| Model 3 |  |  |  |  |  |  |  |  |  |  |  |
| Non-sarcopenia | 1 |  |  |  | 1 |  |  |  | 1 |  |  |
| Sarcopenia | 1.827 | 1.126–2.964 | 0.015 |  | 1.736 | 0.764–3.942 | 0.187 |  | 1.837 | 1.136–2.969 | 0.013 |
| Model 4 |  |  |  |  |  |  |  |  |  |  |  |
| Non-sarcopenia | 1 |  |  |  | 1 |  |  |  | 1 |  |  |
| Sarcopenia | 1.822 | 1.123–2.953 | 0.015 |  | 1.657 | 0.728–3.770 | 0.229 |  | 1.830 | 1.133–2.956 | 0.014 |

Model 1: Age, gender, height, smoking frequency, vitamin D, ALP and PTH levels, FEV_1_ (%), physical inactivity level. Model 2: Model 1 plus weight. Model 3: Model 1 plus BMI. Model 4: Model 1 plus weight and BMI. BMD, bone mineral density; OR, odds ratio; CI, confidence interval; ALP, alkaline phosphatase; PTH, parathyroid hormone; BMI, body mass index.
