# Supplementary material for: Sarcopenia as an Independent Risk Factor for Decreased BMD in COPD Patients: Korean National Health and Nutrition Examination Surveys IV and V (2008-2011)
Source: PLoS One. 2016 Oct 17;11(10):e0164303. doi: 10.1371/journal.pone.0164303 (PMC5066961; doi:10.1371/journal.pone.0164303)
Supplement: S7 Table — (DOCX) [file pone.0164303.s007.docx]

**Table 7**. Odds ratios of osteopenia, osteoporosis, and a low BMD by sarcopenia in each body weight group (subgroup analysis)

|  | Osteopenia | | |  | Osteoporosis | | |  | Low BMD | | |
| --- | --- | --- | --- | --- | --- | --- | --- | --- | --- | --- | --- |
|  | OR | 95% CI | *P* |  | OR | 95% CI | *P* |  | OR | 95% CI | *P* |
| High-weight group | | | | | | | | | | | |
| Non-sarcopenia | 1 |  |  |  | 1 |  |  |  | 1 |  |  |
| Sarcopenia | 2.248 | 1.084–4.665 | 0.030 |  | 4.621 | 1.167–18.291 | 0.029 |  | 2.376 | 1.158–4.877 | 0.018 |
| Low-weight group | | | | | | | | | | | |
| Non-sarcopenia | 1 |  |  |  | 1 |  |  |  | 1 |  |  |
| Sarcopenia | 2.301 | 1.239–4.275 | 0.008 |  | 3.580 | 1.410–9.089 | 0.007 |  | 2.439 | 1.324–4.496 | 0.004 |

Adjusted for age, gender, height, smoking frequency, vitamin D, ALP and PTH levels, FEV_1_ (%), and physical inactivity level. BMD, bone mineral density; AWGS, Asia Working Group for Sarcopenia; OR, odds ratio; CI, confidence interval; ALP, alkaline phosphatase; PTH, parathyroid hormone.
